# Bioeconomic Performance of Pullets and Layer Hens Fed Soybean Grains-Based Diets in Hot and Humid Climate

**DOI:** 10.5402/2012/812564

**Published:** 2012-05-28

**Authors:** M. F. Houndonougbo, C. A. A. M. Chrysostome, F. Daga Dadjo, S. L. Adjaho

**Affiliations:** Faculty of Agronomic Sciences, University of Abomey Calavi, 01 BP 526 Cotonou, Benin

## Abstract

The aim of this paper was to evaluate the effects of toasted soybean grains on bioeconomic performance of pullets and layer hens in hot and humid environment. A total of 972 three-week-old Harco chicks were divided into 12 groups. At starter, pullet and laying phases, birds were fed four diets containing 0% (R0), 5% (R5), 10% (R10), and 15% (R15) of soybean grains. Results showed similar feed intake, body weight gain, laying rate, feed conversion ratio, and mortality rate between dietary treatments at each phase. The egg weight increased significantly in diet R15 (*P* < 0.05). The use of soybean grains reduced the feed prices. Feeding cost decreased significantly (*P* < 0.05) during growth and laying phases in soybean grains added diets. Feeds efficiency increased significantly (*P* < 0.05) with the increase of dietary soybean grains rate. Properly toasted soybean grains can be therefore included up to 15% in heavy line layer hens' diet in tropical conditions.

## 1. Introduction

Soybean meal is an ingredient of choice to supply energy and proteins to layers and broilers. Because of that, costs and availability of soybean meal are strongly correlated with the price of agricultural commodities on the world market [1, 2].

However, soybean grains are more used in broilers diets than layers diets. Soybean grains are known for their high content of fat. The trypsin inhibitors of soybean grain are well characterized and are an important determinant of nutritive value [3, 4]. Properly processed whole soybean grains may be used effectively for poultry [5] and pigs [2]. Toasting is suggested within other heat processing procedures to reduce trypsin inhibitors in soybean grains or meals [6, 7]. In Benin, only Jupiter variety of soybean is produced; and the toasting method is adopted for processing grains and meal [8].

On other side, laying performance of hens is lower when they are too fat. Thus, soybean grains even toasted are used at lower rate in pullets and layers diets than broilers diets. Farmers reject the utilization of toasted soybean grains in layers diet in Benin; but [8] included efficiently up to 22% of toasted soybean grains in broiler diet.

In tropical climate, important increase of dietary energy may be result a decrease of feed intake by poultry. Toasted soybean grains have a high content of energy and protein, it is important to evaluate their optimal rate in diets of pullets and layer hens in hot and humid climate, mainly for birds from heavy lines often used in Africa.

## 2. Materials and Methods

### 2.1. Animals and Housing

The study was conducted in a poultry house (20 m × 15 m). The house was divided into twelve (12) partitions of 25 m^2^ each. Each partition had three feeders (1.5 m of length) and two automatic drinkers.

A total of 1000 Harco (*Rhode Island Red* × *Plymouth Rock*) day-old chicks were imported from Nigeria. They were vaccinated against Newcastle disease, Gumboro, infectious bronchitis, and avian pox. Chicks were also treated regularly against helminthes and coccidiosis.

At three-week-old (starting of the experiment), the average weight of chicks was 206.5 ± 2.69 g. Chicks were divided into 12 groups of 81 chicks each. Thus, at pullet and laying phases, there were 3.2 chicks/m^2^. Each diet was fed in 3 replicates.

### 2.2. Experimental Diets and Feeding

Diets were formulated by phases. At starter (4 to 8 weeks-old), pullet (9 to 18) and laying (19 to 26) phases, respectively, four diets were formulated (Tables 1, 2, and 3). In diets, soybean grains were included at 0% (R0, control), 5% (R5), 10% (R10), and 15% (R15). Soybean grains were toasted before the processing of diets to reduce trypsin inhibitors effect.

At each phase, the same quantity of feed was provided to each replicate and the birds consumed all the available feed. Birds were watered ad libitum. The prices per kg of formulated feed are presented in results. They are used to compare the efficiency of diets.

### 2.3. Statistical Analysis

The general linear model (GLM) was used to analyze data in SAS version 9.1.2 [10]. Mean values are presented in tables with the pooled standard error. A significant effect of diets is stated when *P* value (*P*) is less than 0.05. The effects of replication and of the interaction between diets and replications were not significant (*P* > 0.05). Hence, the statistical model was

(1) $$Y_{i}=\mu +G_{i}+\varepsilon_{i},$$

where *Y*_*i*_ is the observation for dependent variables; *μ* is the general mean; *G*_*i*_ is the fixed effect of soybean grains; *ε*_*i*_ is the residual error.

## 3. Results

The results are presented in two phases: the growth phase (starting and pullet phases) and the laying phase. They relate to feeding, growth, laying, and economic performance. 

### 3.1. Performance of Pullets

**Figure 1:**
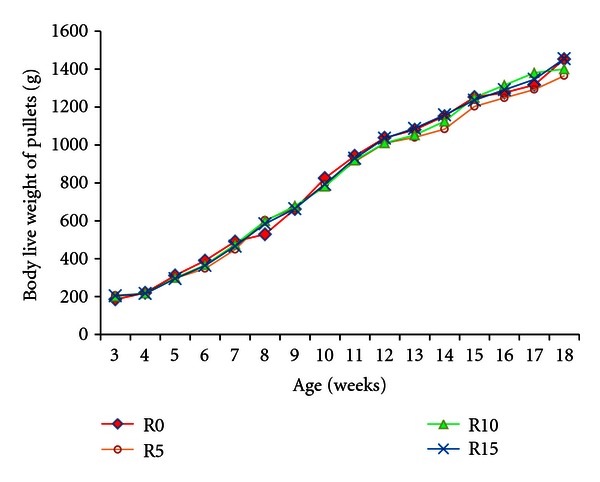
Growth curves of Harco pullets fed different rate of toasted soybean grains based diets. R0, R5, R10, and R15 are diets containing, respectively, 0, 5, 10, and 15% of toasted soybean grains.

Performances during the five weeks of starting phase are shown in Table 4 and Figure 1. The daily feed intake was maintained equal in all diets. No significant difference (*P* > 0.05) was recorded on, daily weight gain, mortality and feed conversion ratio in spite of difference in feed composition between diets. The inclusion of toasted soybean grains at different rates in the diet did not affect significantly the growth and survivability of chicks.

These results suggest a similar efficacy of diets at starter phase. 

Also, during the pullet phase the growth performance was not significantly affected by the diet (Table 5 and Figure 1). However, the feed conversion ratio and the mortality rate increased at pullet compared to the starter phase.

At starter and pullet phases, the prices of the formulated feeds decreased when the soybean grains rate increased in the diet (Table 6). Thus, at pullet phase, the feeding cost per kg of live body weight gain decreased in soybean-based diets compared to the control diet (Table 7). However, at starter phase, due to the light decrease of live body weight gain in the soybean grains-based diets, the feeding cost increased (Table 6). The soybean grains based diets are therefore more efficient at pullet phase than at starter phase.

The feed efficiency evaluated at the end of pullet phase, demonstrated that for each unit of money invested in feed, the revenue from the selling of the live weight gain varied between 2.15 and 2.48 times (Table 7). The efficiency of the diet increased significantly (*P* < 0.05) with an increase of the toasted soybean grains rate. 

### 3.2. Performance of Laying Hens

**Figure 2:**
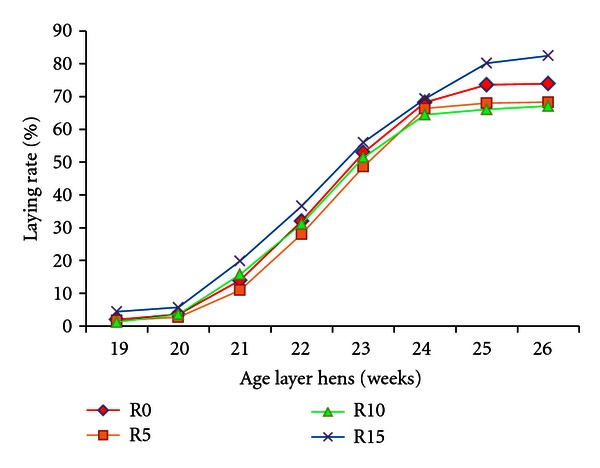
Laying rate of Harco hens fed different rate of toasted soybean grains. R0, R5, R10, and R15 are diets containing, respectively, 0, 5, 10, and 15% of toasted soybean grains.

The laying phase was recorded until the peak of lay between 25 and 26 week-old (Figure 2). While feed intake was equal between treatments, the results demonstrated an improvement of laying rate in layers fed R15 diet compared to the control diet. In the second month, the average laying rate in R15 was 67.8% versus 64.7% in control diet, without any significant difference between treatments. At the peak (26-week-old), laying rates were 73.9%, 68.3%, 67.1%, and 82.5% in R0, R5, R10, and R15 diets, respectively.

Furthermore, the egg weight of layers fed R15 diet increased significantly (*P* < 0.05) in the second month of laying (Table 8). Thus, during the two first months of laying the feed conversion of layers fed R15 was the lowest, and there was no significant effect of diet on mortality rate of hens (*P* > 0.05).

The price of diet was lower in soybean grains diets. Thus, the feeding cost per egg decreased significantly during the first month of laying, but not later (Table 9). Respectively, in first and second months, the feeding cost in R15 diet represented about 32% and 86% of that in control diet, indicating a better efficiency of R15 diet compared to the control diet and the two other soybean grains-based diets. 

## 4. Discussion

### 4.1. Growth Performance of Pullets

The similarity on growth performance of pullets up to 18-week-old demonstrates the efficiency of toasted soybean grains based diets in general and R15 diet in particular. The live body weight gain of pullets was higher than reported [11], and they grew regularly. Thus, the final weights of pullets (1366 to 1456 g) are in the range of 1341 to 1594 g recorded at 20-week-old in Shika-Brown pullets. The live weight of pullets at the starting of the laying period is one of more important criteria focused by farmers. In this study, at 18-week-old the live weight of pullets was very similar in R0 and R15 diets (1454 g versus 1456 g).

During pullet phase the feed conversion ratios were lower than the 5.73 to 6.62 found between 8 and 20 weeks in Hy-line pullets [12]. Furthermore, the light increase of mortality rate in soybean based diets compared to control diet was not significant. These results confirmed the efficacy of all the diets. Up to 15% of toasted soybean grain can be therefore included into pullets' diets in hot and humid climate.

### 4.2. Laying Performance of Hens

Up to peak, the laying rate in R15 diet was the highest, with a significant difference from 24 to 26 weeks of age. Thus, efficiency of toasted soybean grains based diets was also effective during the first eight laying weeks. The average laying rate was higher than 63.8–68.4% [13], 64.0% [14], and 61.4% [12] recorded respectively with Harco and Hy-line layers up to 28-week-old. At laying phase, the feed conversion ratio was also lower in R15 diet compared to the control diet. However, the feed conversion ratios were higher than 2.81–4.07 g feed/g egg [12].

No significant diet effect was found on hens' mortality. In the second month, the egg weights were significantly higher in R15 diet, but lower than the 60 g reported by other [15–17]. In first month, the egg weights were in the range of 41.0–47.4 g [14].

Energy requirement of hens is lower in hot and humid climate than in temperate climate. Soybean grains being very energetic, during the latest laying weeks, hens could get fat. That might reduce their laying performance. An evaluation of the laying performance during the whole laying period is therefore relevant for heavy layers breeds fed with whole toasted soybean grains in hot and humid climate.

### 4.3. Economic Performance of Pullets and Hens

The incorporation of toasted soybean grains in diets reduces feed prices. At growth and laying phases, the lowest feeding cost was in R15 diet. Thus, feeding cost and feed efficiency improved significantly in soybean-based diets during the growth phase of pullets. The feeding of Harco pullets with toasted soybean grains diets can be therefore recommended in hot and humid regions. The significant decrease of feeding cost from first to second laying months was due to the increase of laying rate until the peak.

## 5. Conclusion

This study shows the efficiency of toasted soybean-based diets in Harco pullets and hens feeding in hot and humid climate. The toasting processing used in Benin to improved soybean grain efficacy in poultry diet is therefore suitable. However, the price of toasted soybean grains should be kept at a level where the energy and protein costs from these grains should be lower than those from other main energy and protein sources like soybean and fish meals. 

## Figures and Tables

**Table 1:** Ingredients and chemical composition of diets as formulated at starter phase (4- to 8-week-old).

Ingredients (%)	R0	R5	R10	R15
Soybean grains^1^	—	5	10	15
Maize grain	60.35	55.35	54.35	53.35
Wheat bran	13	19	19	19
Soybean meal	15	—	—	—
Cotton meal^2^	5	10	8	6
Oyster shell	1	1	1	1
Lysine	0.1	0.1	0.1	0.1
Methionine	0.2	0.2	0.2	0.2
Phosphate bicalcium	0.1	0.1	0.1	0.1
Premix^3^	0.25	0.25	0.25	0.25
Concentrated broilers	5	9	7	5

Chemical composition				
Dry matter (%)	86.5	86.9	86.8	86.7
Crude fiber (%)	4.3	4.6	4.7	4.7
Metabolisable energy (kcal/kg)	2761.2	2717.8	2793	2868
Crude protein (%)	17.8	17.6	17.6	17.7
Lysine (%)	0.92	0.87	0.89	0.91
Methionine (%)	0.56	0.61	0.58	0.55
Methionine + cystine (%)	0.85	0.88	0.86	0.85
Calcium (%)	0.84	1.10	0.97	0.83
Total phosphorus (%)	0.75	0.96	0.88	0.80

^1^Toasted soybean grains made in Benin.

^2^Ferrous sulphate (FeSO_4_) was added at the rate of 3 g per kg of cotton meal.

^3^Premix contained per kg: vitamins: A 4000000 UI, D3 800000 UI, E 2000 mg, K 800 mg, B1 600 mg, B2 2000 mg, niacin 3600 mg, B6 1200 mg, B12 4 mg, and choline chloride 80000 mg; minerals: Cu 8000 mg, Mn 64000 mg, Zn 40000 mg, Fe 32000 mg, and Se 160 mg.

**Table 2:** Ingredients and chemical composition of diets as formulated at pullet phase (9- to 18-week-old).

Ingredients (%)	R0	R5	R10	R15
Soybean grains^1^	—	5	10	15
Maize grain	56.15	60.15	52.15	51
Wheat bran	7.6	6.6	9.6	11.6
Soybean meal	14	—	—	—
Cotton meal^2^	7	10	10	7
Oyster shell	1.7	1.7	1.7	1.7
Lysine	0.1	0.1	0.1	0.1
Methionine	0.2	0.2	0.2	0.2
Phosphate bicalcium	1	1	1	1
Premix^3^	0.25	0.25	0.25	0.25
Concentrate broilers	5	5	5	5
Maize bran	7	10	10	7.15

Chemical composition				
Dry matter (%)	87.2	87.2	87.3	87.2
Crude fiber (%)	4.63	4.45	4.93	5.13
Metabolisable energy (kcal/kg)	2819	2816	2817	2847
Crude protein (%)	15.92	15.39	15.88	15.75
Lysine (%)	0.78	0.72	0.75	0.76
Methionine (%)	0.37	0.43	0.39	0.36
Methionine + cystine (%)	0.66	0.69	0.67	0.65
Calcium (%)	0.91	1.23	1.04	0.91
Total phosphorus (%)	0.64	0.82	0.74	0.67

^1^Toasted soybean grains made in Benin.

^2^Ferrous sulphate (FeSO_4_) were added at the rate of 3 g per kg of cotton meal.

^3^Premix contained per kg: vitamins: A 4000000 UI, D3 800000 UI, E 2000 mg, K 800 mg, B1 600 mg, B2 2000 mg, niacin 3600 mg, B6 1200 mg, B12 4 mg, and choline chloride 80000 mg; minerals: Cu 8000 mg, Mn 64000 mg, Zn 40000 mg, Fe 32000 mg, and Se 160 mg.

**Table 3:** Ingredients and chemical composition of diets as formulated at laying phase (19- to 26-week-old).

Ingredients (%)	R0	R5	R10	R15
Soybean grains^1^	—	5	10	15
Maize grain	57.75	57.25	54.25	59.75
Wheat bran	—	—	2.5	—
Soybean meal	19	14.5	9.5	—
Cotton meal^2^	7	7	7.5	9
Oyster shell	10	10	10	10
Lysine	0.05	0.05	0.05	0.05
Methionine	0.15	0.15	0.15	0.15
Phosphate bicalcium	0.8	0.8	0.8	0.8
Premix^3^	0.25	0.25	0.25	0.25
Concentrate layers	5	5	5	5

Chemical composition				
Dry matter (%)	88.7	88.5	88.5	88.4
Crude fiber (%)	3.55	3.53	3.7	3.28
Metabolisable energy (kcal/kg)	2703	2702	2708	2814
Crude protein (%)	18.2	18.2	18.2	18.55
Lysine (%)	0.93	0.93	0.9	0.93
Methionine (%)	0.51	0.51	0.50	0.59
Methionine + cystine (%)	0.80	0.80	0.80	0.87
Calcium (%)	4.38	4.38	4.4	4.60
Total phosphorus (%)	0.75	0.74	0.8	0.83

^1^Toasted soybean grains made in Benin.

^2^Ferrous sulphate (FeSO_4_) were added at the rate of 3 g per kg of cotton meal.

^3^Premix contained per kg: vitamins: A 4000000 UI, D3 800000 UI, E 2000 mg, K 800 mg, B1 600 mg, B2 2000 mg, niacin 3600 mg, B6 1200 mg, B12 4 mg, and choline chloride 80000 mg; minerals: Cu 8000 mg, Mn 64000 mg, Zn 40000 mg, Fe 32000 mg, and Se 160 mg.

**Table 4:** Feeding and growth performance of pullet chicks from 4 to 8 weeks of age.

	R0^1^	R5	R10	R15	SE	*P* ^3^
Daily feed intake (g/day)	41	41	41	41	—	—
Final live body weight (g)	529.4	603.1	599.3	564.5	36.1	0.75
Daily weight gain (g/day)	12.7	11.34	11.38	10.84	1.56	0.85
Mortality rate (%)	0.00	0.25	0.25	0.08	0.10	0.23
Feed conversion ratio^4^	1.74	1.86	1.81	1.84	0.13	0.92

^1^R0, R5, R10, and R15 are diets containing respectively 0, 5, 10, and 15% of toasted soybean grains.

^2^Standard error; ^3^ *P* value; ^4^kg feed/kg live body weight gain.

**Table 5:** Feeding and growth performance of pullets from 9 to 18 weeks of age.

	R0^1^	R5	R10	R15	SE	*P* ^3^
Daily feed intake (g/day)	78.0	78.0	78.0	78.0	—	—
Final live body weight (g)	1454	1366	1401	1456	29.9	0.73
Daily weight gain (g/day)	13.9	13.4	13.9	14.6	1.36	0.74
Mortality rate (%)	0.16	0.38	0.35	0.69	0.14	0.08
Feed conversion ratio^4^	3.69	3.82	3.75	3.69	0.11	0.8

^1^R0, R5, R10, and R15 are diets containing respectively 0, 5, 10, and 15% of toasted soybean grains.

^2^Standard error; ^3^ *P*-value; ^4^kg feed/kg live body weight gain.

**Table 6:** Economic performance during the starter phase (4 to 8 weeks of age).

	R0^1^	R5	R10	R15	SE	*P* ^3^
Price of formulated feed (FCFA*/kg)	234	227	223	219	—	—
Feeding cost (FCFA/kg body weight gain)	877.1^a^	1383.4^b^	1174.8^c^	1459.8^b^	866	0.02

^a, b, c^ Means with unlike superscripts in the same row differ significantly (*P* < 0.05).

^1^R0, R5, R10, and R15 are diets containing respectively 0, 5, 10, and 15% of toasted soybean grains.

^2^Standard error; ^3^ *P*-value; *1*€* = 655.9 FCFA.

**Table 7:** Economic performances during pullet phase (9 to 18 weeks of age).

	R0^1^	R5	R10	R15	SE	*P* ^3^
Price of formulated feed (FCFA*/kg)	202	206	197	192	—	—
Feeding cost (FCFA/kg weight gain)	1908.4^a^	1677.6^b^	1649.5^b^	1323.7^c^	1079	0.04
Economic feed efficiency^4^	2.37^a^	2.15^b^	2.30^a^	2.48^c^	0.08	0.03

^a, b, c^ Means with unlike superscripts in the same row differ significantly (*P* < 0.05).

^1^R0, R5, R10, and R15 are diets containing respectively 0, 5, 10, and 15% of toasted soybean grains.

^2^Standard error; ^3^ *P*-value; *1*€* = 655.9 FCFA.

^4^(FCFA Body weight gain/FCFA feed): revenue from weight gain/feeding cost, according to [9].

**Table 8:** Feeding and laying performances of layer hens fed different rate of toasted soybean grains between 19- and 26-week-old.

	Laying months	R0^1^	R5	R10	R15	SE	*P* ^3^
Daily feed intake (g/day)	1	113.8	113.8	113.8	113.8	—	—
	2	120	120	120	120	—	—
Egg weight (g)	1	44.9	46.1	45.1	44.1	1.28	0.78
	2	52.0^a^	51.4^a^	52.4^a^	55.1^b^	0.72	0.004
Mortality rate (%)	1	0.31	0.22	0.55	0.11	0.19	0.44
	2	1.09	0.89	0.56	1.15	0.31	0.56
Feed conversion ratio (kg feed/kg egg)	1	20.3^a^	26.7^b^	20.9^a^	16.9^a^	1.84	0.005
	2	7.16^a^	7.97^b^	7.88^b^	6.36^a^	0.37	0.014

^a, b^Means with unlike superscripts in the same row differ significantly (*P* < 0.05).

^1^R0, R5, R10, and R15 are diets containing, respectively 0, 5, 10, and 15% of toasted soybean grains.

^2^Standard error; ^3^ *P*-value.

**Table 9:** Economic performances of layer hens fed different rate of toasted soybean grains.

	Laying month	R0^1^	R5	R10	R15	SE	*P* ^3^
Feed price (FCFA*/kg)	**—**	270	260	255	260	**—**	**—**
Feeding cost (FCFA/Egg)	1	2142.7^a^	1930.9^a^	1700.5^a^	1652.8^b^	681.0	0.04
	2	54.8	57.2	55.2	47.3	4.21	0.37

^a, b^Means with unlike superscripts in the same row differ significantly (*P* < 0.05).

^1^R0, R5, R10 and R15 are diets containing respectively 0, 5, 10, and 15% of toasted soybean grains.

^2^Standard error; ^3^ *P*-value;

*1*€* = 655.9 FCFA.
